# Identification of Cold-Responsive miRNAs and Their Target Genes in Nitrogen-Fixing Nodules of Soybean

**DOI:** 10.3390/ijms150813596

**Published:** 2014-08-05

**Authors:** Senlei Zhang, Youning Wang, Kexue Li, Yanmin Zou, Liang Chen, Xia Li

**Affiliations:** 1 The State Key Laboratory of Plant Cell & Chromosome Engineering, Center of Agricultural Research Resources, Institute of Genetics and Developmental Biology, Chinese Academy of Sciences, 286 Huaizhong Road, Shijiazhuang, Hebei 050021, China; E-Mails: lovemyfish8@126.com (S.Z.); youningwang@163.com (Y.W.); kexuely@163.com (K.L.); ymzou@genetics.ac.cn (Y.Z.); cl007z@aliyun.com (L.C.); 2 College of Life Sciences, University of Chinese Academy of Sciences, 19A Yuquan Road, Beijing 100049, China

**Keywords:** microRNAs, functional nodules, low temperature, *Glycine max* (L.) Merrill

## Abstract

As a warm climate species, soybean is highly sensitive to chilling temperatures. Exposure to chilling temperatures causes a significant reduction in the nitrogen fixation rate in soybean plants and subsequent yield loss. However, the molecular basis for the sensitivity of soybean to chilling is poorly understood. In this study, we identified cold-responsive miRNAs in nitrogen-fixing nodules of soybean. Upon chilling, the expression of gma-miR397a, gma-miR166u and gma-miR171p was greatly upregulated, whereas the expression of gma-miR169c, gma-miR159b, gma-miR319a/b and gma-miR5559 was significantly decreased. The target genes of these miRNAs were predicted and validated using 5' complementary DNA ends (5'-RACE) experiments, and qPCR analysis identified putative genes targeted by the cold-responsive miRNAs in response to chilling temperatures. Taken together, our results reveal that miRNAs may be involved in the protective mechanism against chilling injury in mature nodules of soybean.

## 1. Introduction

Cold stress, including chilling (<20 °C) and freezing (<0 °C), influences plant development and crop yields. The genetic and physiological mechanisms underlying chilling injury and tolerance are well understood in *Arabidopsis*. In order to adapt to cold stress, plants alter their metabolism and growth through reprogramming of gene expression [1,2]. The perception of a cold signal in plants leads to the activation of C-repeat/drought-responsive element binding factor (CBF)-dependent and CBF-independent transcriptional pathways that modulate the response to cold stress [3,4].

Numerous studies conducted in a wide range of species have shown that microRNA (miRNA)-based gene regulation is essential for coordinating plant responses to cold stress [5,6,7]. In *Arabidopsis*, the expression of miR165/166, miR319c and miR169 is induced by cold stress [8,9], while in *Brachypodium*, the expression of both miR172 and miR397 is up-regulated by cold treatment [10]. The reported targets of miR166 are *PHB* (*At2g34710*) and *ATHB-8* (*At4g32880*), which are down-regulated by cold exposure [8]. Recently, an analysis of deep sequencing data from a wheat thermosensitive genic male sterile line showed that 78 unique miRNAs may play a regulatory role in male sterility during cold stress [11]. miR319 has also been shown to regulate cold responses in both sugarcane [12] and *Populus* [13].

Soybean (*Glycine max*) is a globally important crop and a key source of proteins for human and animal consumption. Soybean is also used as a natural nitrogen source in agriculture, because nitrogen-fixing bacteria in specialized organs, called “root nodules”, can effectively convert atmospheric N_2_ into ammonium [14]. It has been reported that symbiotic nitrogen fixation (SNF) is highly sensitive to abiotic stresses, including defoliation, drought, extended dark treatment and chilling [15,16,17]. Notably, soybean is a warm climate crop species; thus, its growth and productivity are sensitive to chilling temperatures. For example, when subjected to even a single low temperature below 15 °C (also known as dark chilling), nitrogen fixation rates can drop up to 45% and pod formation is completely inhibited, leading to a significant loss of plant productivity [16,18]. In the past two decades, we have made great progress in elucidating the physiological mechanisms involved in plant responses to chilling, including photosynthesis, carbon metabolism and nitrogen fixation [16,19,20]. However, our understanding of the molecular mechanisms that modulate plant responses to chilling remains limited. In particular, the molecular mechanisms by which nitrogen-fixing nodules respond to chilling and modulate SNF in soybean are elusive.

Recently, a genome-wide analysis of miRNAs revealed key regulatory roles of miRNAs in nodulation, including *Bradyrhizobium japonicum* infection, nodule formation and nitrogen-fixation [21,22,23,24,25,26]. However, there is no report on the roles of miRNAs and their target genes in SNF in response to low temperatures in the nitrogen-fixing nodules of soybean. In this study, we performed a deep sequencing analysis of miRNAs in nitrogen-fixing soybean nodules to determine the expression profile of miRNAs in response to cold. Eleven miRNAs that were highly responsive to cold treatment were identified. The putative target genes of the miRNAs were predicted, and the predicted cleavage sites were verified *in vivo* for a subset of these targets by rapid amplification of cDNA ends (5'-RACE, 5' complementary DNA ends) analysis. Based on these results, we found that in cold-treated soybean plants, miR166u, miR171p, miR2111f and miR169c may regulate different targets in mature nodules through mRNA degradation. Our results have important implications for miRNA regulation in mature nodules in response to low temperatures and the modulation of SNF.

## 2. Results and Discussion

### 2.1. Results

#### 2.1.1. The Nitrogen Use Efficiency of Nitrogen-Fixing Nodules Was Decreased in Response to Low Temperature Treatment

**Figure 1:**
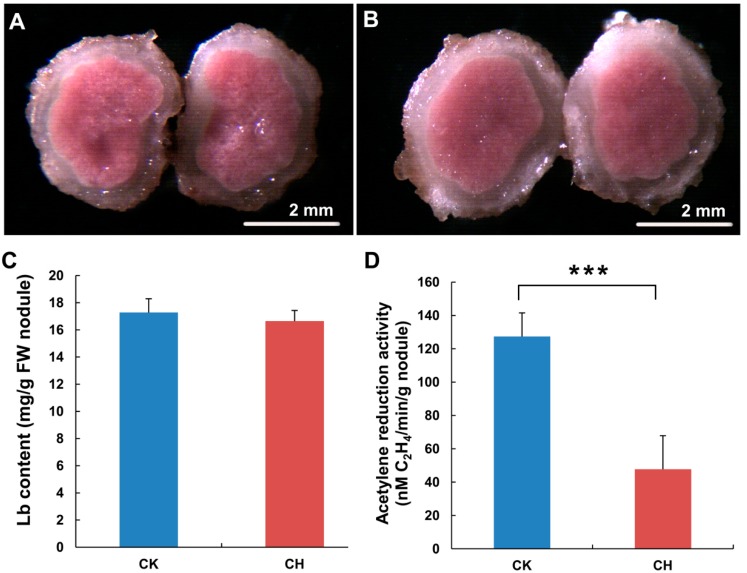
The nitrogen use efficiency of nitrogen-fixing nodules in soybean was affected by low temperature treatment. Seeds of Williams 82 were sown into a mixture of vermiculite and perlite and irrigated with a low nitrogen solution. Twenty-eight days after inoculation with *Bradyrhizobium japonicum*, the plants were dipped and washed with phosphate-buffered saline and then transferred to a low nitrogen solution without (CK) or with cold treatment (CH) at 4 °C for 24 h. The nodules were then collected and used for a phenotypic analysis and measurements of the leghemoglobin (Lb) content and acetylene reduction activity (ARA). Cross-sections of untreated and cold-treated nodules are shown in (**A**,**B**), respectively. Bars: 2 mm; The measured Lb contents and ARA levels are shown in (**C**,**D**), respectively. Student’s *t*-tests were performed; statistically significant results are marked with *** (*p* < 0.001).

To investigate the effect of low temperatures on the nitrogen-fixing ability of mature nodules, we first measured the leghemoglobin (Lb) content and nitrogenase activity (measured as acetylene (C_2_H_2_) reduction activity, or ARA) at 28 days after rhizobium inoculation in nitrogen-fixing nodules treated at 4 °C for 24 h. Lb is bright red in color, and Lb accumulation or nodule coloration is considered to be an indicator of the maturity and functionality of root nodules [27]. It has been shown that the Lb content of plant nodules is adversely affected by various abiotic stresses, including drought, cadmium, sodium and nitrite, and by senescence in many legumes, such as *Lupinus albus*, *Pisum sativum*, *Medicago truncatula*, *Lotus japonicus* and soybean [28,29,30,31,32,33]. Lowering of the Lb level is mainly due to the degradation and/or heme nitration of Lb, leading to a color change from pink to green [34,35]. As shown in Figure 1A,B, there was no apparent difference in nodule color between the cold-treated and untreated nodules. The Lb content of the cold-treated nodules was also not significantly changed compared with that of the untreated control nodules (Figure 1C).

Next, the ARA of nitrogenase was analyzed using gas chromatography as described by Boyd [36]. Intriguingly, we found that nitrogenase activity in the functional nodules was markedly decreased by approximately 60% 24 h after chilling treatment (Figure 1D). Nitrogenases are used by rhizobia to fix atmospheric N_2_, and their enzymatic activity is directly correlated with nitrogen fixation efficiency [37]. Our results therefore suggest that the nitrogen-fixing ability of mature nodules is influenced by chilling treatment. Previous studies have shown that nitrogenase activity is sensitive to various environmental stresses, including drought, heat and nitrite [37,38,39,40,41]. Our results indicate that the reduced nitrogen fixing efficiency of the nodules caused by chilling was mainly due to decreased nitrogenase activity, rather than the Lb content.

#### 2.1.2. Identification and Validation of Low Temperature-Responsive miRNAs in Functional Nodules

To explore whether there was any connection between miRNA expression and the decrease in nitrogenase activity, we used Solexa sequencing technology to analyze the miRNAs in cold-treated (CH) and untreated (CK) mature nodules (Figure S1 and Tables S1 and S2). Equal amounts of total RNA from CK and CH were pooled and used to construct two small regulatory RNA (sRNA) libraries to augment the chance of finding as many miRNAs as possible in a single experiment. Overall, more than 33 million raw reads were obtained from the two libraries. Clean reads (excluding reads smaller than 18 nucleotides (nt) and adaptors) accounted for around 89% of the total (Table S2). For unique sRNAs (*i.e*., sRNAs with a unique sequence), more than 42% of the reads from the CK library and more than 35% of the reads from the CH library could be mapped to the soybean genome, respectively (Table S2).

**Figure 2:**
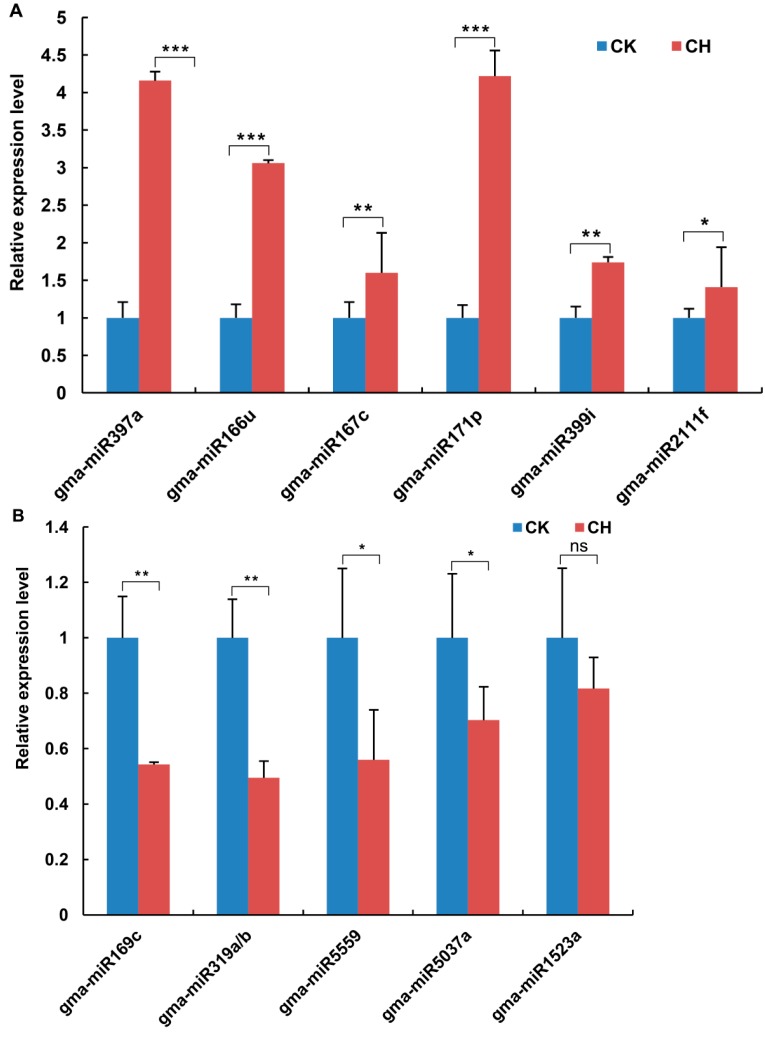
Quantitative reverse transcription PCR (RT-qPCR) analysis of miRNA expression in low temperature-treated mature nodules. Small RNAs were isolated from functional soybean nodules 28 days after inoculation with *B. japonicum* strain USDA110 in the cold (4 °C for 24 h); untreated nodules were used as a control. (**A**) The miRNAs that were up-regulated by low-temperature treatment are shown; (**B**) The miRNAs that were down-regulated by low-temperature treatment are shown. 5.8S rRNA was used as an internal control. The results shown are the averages ± SE of three biological replicates. Error bars indicate the standard deviation. Student’s *t*-tests were performed; statistically significant results are marked with * (*p* < 0.05); ** (*p* < 0.01); *** (*p* < 0.001); ns: not significant (*p* > 0.05).

We found that 61 miRNAs belonging to known miRNA families were responsive to low-temperature treatment. To validate our sequencing results, we performed quantitative reverse transcription PCR (RT-qPCR) to analyze the expression of the miRNAs in nitrogen-fixing nodules with or without low temperature treatment. We found that the expression level of eleven miRNAs was highly affected by low temperature treatment. These miRNAs were used for further study. As shown in Figure 2, six miRNAs were clearly upregulated by low-temperature treatment in mature nodules (Figure 2A). Among them, the expression levels of gma-miR397a, gma-miR166u and gma-miR171p were increased more than three-fold, whereas the expression of gma-miR167c, gma-miR399 and gma-miR2111f showed a fractional increase (Figure 2A). The rest of the miRNAs were downregulated by different degrees in low temperature-treated nitrogen-fixing nodules (Figure 2B). The expression levels of gma-miR169c, gma-miR159b and gma-miR319a/b were reduced by approximately 50%. These results suggest that miRNAs and miRNA-mediated molecular pathways modulate nitrogen fixation in low temperature-treated nodules.

To obtain additional information about the potential roles of miRNAs in nitrogen fixation at low temperatures, we analyzed the promoters (2 kb in length) of the miRNAs, gma-miR166u, gma-miR171p and gma-miR2111f, using plantPAN [42]. Interestingly, in addition to *cis* elements for binding auxin response factors, we found a binding site (MYCCONSENSUSAT) for inducer of CBF expression 1 (ICE1) and *cis* elements for binding cold-responsive transcription factors, such as RAV1. Taken together, our results reveal novel roles for these miRNAs in regulating the response of nitrogen-fixing nodules to low temperatures (Figures S2 and S3).

#### 2.1.3. Target Prediction and Validation for the Identified Low Temperature-Responsive miRNAs

miRNAs regulate gene expression post-transcriptionally by promoting mRNA decay or by inhibiting translation [24]. To identify candidate target genes of our low temperature-responsive miRNAs, we used two prediction algorithms: psRNAtarget [43] and the target search tool in WMD3 [44]. As shown in Table 1, 48 genes were predicted to be targets of the 11 low temperature-responsive miRNAs (Table 1). These putative target genes encode a variety of proteins, including a multicopper oxidase, putative lysophospholipase, transporters and transcription factors, indicating that the miRNAs play multiple regulatory roles in the cold response of soybean.

**Figure 3:**
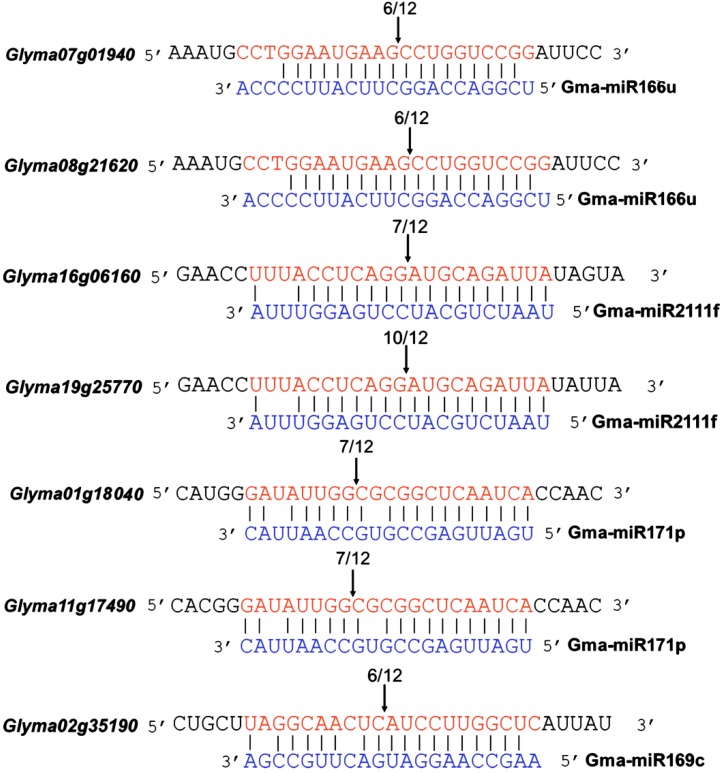
Validation of the miRNA-directed cleavage of the putative target genes using rapid amplification of 5' complementary DNA ends (5'-RACE). The predicted targets of *gma-miR171p*, *gma-miR166u*, *gma-miR2111f* and *gma-miR169c* were experimentally validated. The mRNA cleavage sites were determined by modified 5' RNA ligase-mediated (5'-RLM)-RACE. The mRNA sequence of each complementary site and its 5' and 3' flanking sequences (5 nucleotides (nt) from 5' to 3') and cloned miRNA sequence (from 3' to 5') are shown. Vertical arrows indicate the 5' termini of the miRNA-guided cleavage products, as identified by 5'-RACE, with the frequency of clones shown.

**Figure 4:**
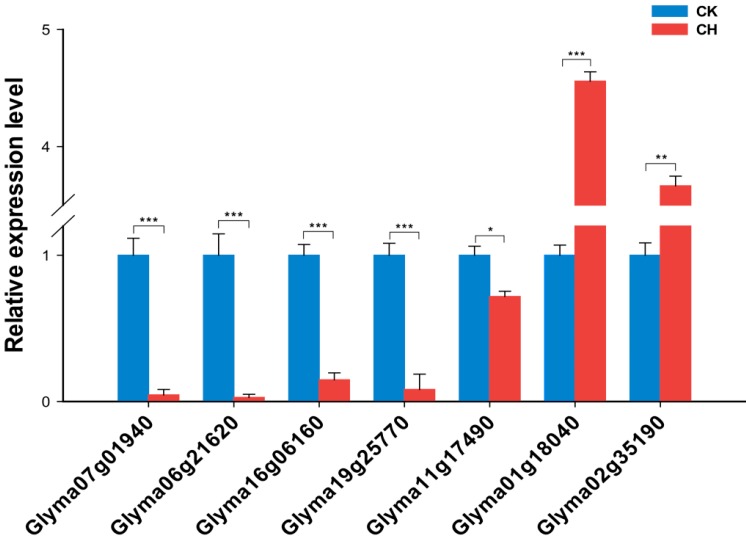
Expression levels of the validated target genes in low temperature-treated mature nodules. Total mRNA from cold-treated or untreated mature nodules was extracted, and RT-qPCR was used to analyze the relative expression of the selected target genes, including *glyma07g01940* and *glyma08g21620* targeted by gma-miR166u, *glyma16g06160* and *glyma19g25770* targeted by gma-miR2111f, *glyma11g17490* and *glyma01g18100* targeted by gma-miR171p and *glyma02g35190* targeted by gma-miR169c. *ELF1b* was used as an internal control. The results given are averages ± SE of three biological replicates. Error bars indicate the standard deviation. Student’s *t*-tests were performed; statistically significant results are marked with * (*p* < 0.05); ** (*p* < 0.01); and *** (*p* < 0.001).

To validate whether these genes are directly targeted by the corresponding miRNAs, we performed a 5'-RACE assay to confirm the cleavage of the predicted target genes by miR166u, miR2111f, miR171p and miR169c (Figure 4). We found that all of the predicted target genes encoding basic leucine zipper (bZIP) or GAI-RGA-SCR (GRAS) family transcription factors (*glyma11g17490* and *glyma01g18040* for gma-miR171p and *glyma07g01940* and *glyma08g21620* for of gma-miR166u) were cleaved at a high frequency (Figure 3). In addition, two genes, *glyma16g06160* and *glyma19g25770*, encoding Kelch-related proteins, were validated as targets of miR2111f. *Glyma02g35190*, which encodes a HAP2-like transcription factor, was also directly cleaved by gma-miR169c. All of the target genes were cleaved by only one miRNA at a single cleavage site. While the target genes of gma-miR166u and gma-miR2111f were cleaved at the expected sites (between the 10th and 11th nucleotides (nt) from the complementary 5' end of the miRNAs), we found that the cleavage sites of the target genes for gma-miR171p and gma-miR169c were located between the 13th/14th and 11th/12th nt, respectively.

We also analyzed the promoters of the validated target genes and found multiple *cis* elements for binding cold-responsive transcription factors (e.g., ICE1) and auxin response factors (Figure S2). Our findings indicate that these genes are functional targets of the miRNAs in cold-treated soybean.

**Table 1:** Putative target genes of known cold-responsive miRNAs and their function annotations.

miRNA	Target Gene	Target Annotation
gma-miR397a	Glyma01g26750 ^a,b^	Multicopper oxidase, oxidation/reduction
	Glyma18g07240 ^b^	Multicopper oxidase, oxidation/reduction
	Glyma03g15800 ^a,b^	Multicopper oxidase, oxidation/reduction
	Glyma03g14450 ^b^	Multicopper oxidase, oxidation/reduction
	Glyma06g43700 ^b^	Multicopper oxidase, oxidation/reduction
	Glyma12g14230 ^b^	Multicopper oxidase, oxidation/reduction
gma-miR166u	Glyma08g21620 ^a,c^	bZIP transcription factor
	Glyma07g01940 ^a,c^	Homeobox-leucine zipper protein
gma-miR171p	Glyma01g18040 ^a,b,c^	GRAS family transcription factor
	Glyma11g17490 ^a,c^	GRAS family transcription factor
gma-miR2111f	Glyma19g01430 ^a,b^	EamA-like transporter family
	Glyma15g34831 ^a,b^	EamA-like transporter family
	Glyma16g06160 ^b,c^	Kelch motif; Protin Binding; Kelch-related protein
	Glyma19g25770 ^b,c^	Kelch motif; Protin Binding; Kelch-related protein
gma-miR169c	Glyma02g35190 ^a,b,c^	HAP2 like transcription factor
	Glyma19g38800 ^a,b^	HAP2 like transcription factor
	Glyma13g16770 ^a,b^	HAP2 like transcription factor
	Glyma07g04050 ^a,b^	HAP2 like transcription factor
	Glyma13g27230 ^a,b^	HAP2 like transcription factor
gma-miR5037a	Glyma17g13680 ^a,b^	GRAS family transcription factor
	Glyma05g03020 ^a,b^	GRAS family transcription factor
gma-miR167c	Glyma11g31940 ^a,b^	Auxin response factor
	Glyma18g05330 ^a,b^	Auxin response factor
	Glyma02g40650 ^a,b^	Auxin response factor
	Glyma14g03650 ^a,b^	Auxin response factor
	Glyma14g38940 ^a,b^	Auxin response factor
	Glyma08g10550 ^a,b^	Auxin response factor
gma-miR319a/b	Glyma13g04030 ^a,b^	MYB family transcription factor
	Glyma13g25720 ^a,b^	MYB family transcription factor
	Glyma15g35860 ^a,b^	MYB family transcription factor
	Glyma20g11040 ^a,b^	MYB family transcription factor
	Glyma06g43720 ^a,b^	TCP family transcription factor
	Glyma12g14200 ^a,b^	TCP family transcription factor
gma-miR399i	Glyma10g04230 ^a,b^	Inorganic phosphate and sugar transporter
	Glyma14g36650 ^b^	Inorganic phosphate and sugar transporter
	Glyma19g34710 ^a^	Inorganic phosphate and sugar transporter
	Glyma20g34620 ^a^	Inorganic phosphate and sugar transporter
gma-miR1523a	Glyma13g25040 ^b^	Putative lysophospholipase
	Glyma13g25050 ^b^	Putative lysophospholipase
gma-miR5559	Glyma20g34670 ^b^	Mediator complex subunit 28
	Glyma02g19340 ^b^	No functional annotation
	Glyma14g02030 ^b^	Protein binding
	Glyma02g46640 ^b^	Protein binding

^a^ predicted by psRNAtarget; ^b^ predicted by WMD3; ^c^ miRNA targets validated by RLM-5'-RACE.

#### 2.1.4. Correlation between the Expression of miRNAs and Their Targets in Response to Low Temperature Stress in Mature Nodules

If a gene acts as a functional target of a miRNA in regulating the cold response of nitrogen-fixing nodules, it should show an opposite pattern of expression to the miRNA in response to low temperatures. To this end, we analyzed the expression patterns of our validated miRNA targets in low temperature-treated nitrogen-fixing nodules using RT-qPCR. As shown in Figure 4, the expression levels of the target genes were negatively correlated with the corresponding miRNAs. Importantly, the expression of *glyma07g01940*, *glyma06g21620*, *glyma16g06160* and *glyma19g25770* was greatly repressed by cold treatment (Figure 4), in contrast to the corresponding miRNAs (Figure 2). The expression levels of gma-miR169c and *glyma02g35190* were also negatively correlated (Figure 4). Interestingly, an exception was noted for the validated target genes, *glyma11g17490* and *glyma01g18040* (Figure 3). Of these, *glyma11g17490* showed an opposite expression pattern to gma-miR171p, whereas *glyma01g18040* was highly induced by cold treatment. These data suggest that *glyma11g17490* is the direct target of gma-miR171p in the regulation of cold-induced nodular changes.

### 2.2. Discussion

The recent genome-wide identification of miRNAs in low temperature-treated plants revealed the critical regulatory roles of miRNAs in plant responses to cold stress [5,6,7]. However, evidence for the miRNA-based regulation of cold responses in symbioses between plants and other organisms (e.g., bacteria) is lacking. It has been shown that miRNAs are involved in the establishment of legume-rhizobia symbioses, the functionality of mature nodules and the response to salt stress [22,45,46,47]. As a warm climate species, soybean exhibits high rates of nodulation and nitrogen fixation. The nitrogen fixation efficiency of soybean is substantially reduced at suboptimal temperatures [16,18]. Here, we demonstrated that the level of nitrogenase activity in functional nodules was decreased by more than 60% within 24 h of low temperature treatment (Figure 1D). Our results support the notion that low temperatures lead to a decreased metabolism in soybean-rhizobia symbioses and an immediate reduction in nitrogenase activity. Understanding the molecular mechanisms of temperature sensing and the signal transduction pathways in soybean-rhizobia symbioses, especially the nitrogen fixation function of nodules, will facilitate the improvement of the nitrogen fixation efficiency of soybean. In this study, we identified eleven low temperature-responsive miRNAs. We also validated the target genes for four miRNAs, and, more importantly, we identified the genes targeted by the miRNAs in nodules in response to cold. To our knowledge, this is the first report on the role of miRNAs in nodules and nitrogen fixation in response to low temperatures.

Among the eleven cold-responsive miRNAs we identified, four (miR166, miR171, miR2111 and miR169) are highly conserved in higher plants, and these miRNAs have been shown to regulate plant cold responses [8,10,11,48,49]. Interestingly, the expression patterns of these miRNAs vary in response to low temperatures in different plants. For example, miR169 is upregulated under cold stress in *Arabidopsis*, *Brachypodium* and rice [8,10,48,50]. By contrast, some specific members of the miR169 family are downregulated by cold treatment (e.g., miR169f/j/h/i in *Populus* and miR169d in *Poncirus*) [49,51]. Such findings indicate that these miRNAs or members of the miRNA family are involved in plant cold responses, but may have acquired diverse functions during evolution. Further, we found that miR169c expression was repressed by low-temperature treatment in nitrogen-fixing nodules (Figure 2B). Therefore, we propose that the conserved cold-responsive miRNAs and their mediated pathways play a key role in the cold responses of nitrogen-fixing nodules and soybean-rhizobia symbioses. Notably, previous studies have shown that miR166, miR171, miR2111 and miR397 are involved in nodulation in the legumes, *Medicago* and *Lotus* [52,53,54,55]. For example, *Lotus* miR171 and miR397 modulate rhizobial infection by targeting a transcription factor gene, *Nodulation Signaling Pathway 2*, and a gene encoding a laccase copper protein, respectively [53,56]. Thus, multiple miRNAs, including miR166, miR171, miR2111 and miR397, are required for nodule development, nitrogenase activity and nitrogen fixation efficiency control in nodules and in the adaptation of plants to adverse environmental conditions.

Our results also show that miRNAs are master regulators of the response of nodules to low temperatures. The miRNAs identified in this study target genes encoding transcription factors, enzymes and transporters that are involved in many biological processes, including the activation/repression of downstream genes, oxidation and reduction and the transportation of organic/inorganic molecules related to nitrogenase activity and nodule function (Table 1). miR166 is predicted to target homeodomain (HD)-ZIP transcription factors, and the target sites are conserved in *Arabidopsis*, rice, *Brachypodium*, maize and *Populus* [9,10,13,49,57,58]. In soybean, we found that *Glyma08g21620* and *Glyma07g01940* were cleaved by gma-miR166u (Figure 3), and both genes showed opposite expression patterns in response to cold (Figure 4). *Glyma08g21620* and *Glyma07g01940* encode a basic leucine zipper (bZIP) transcription factor and an HD-ZIP protein, respectively. Because bZIP and HD-ZIP transcription factors have diverse roles in plant development and stress responses [59,60,61], we speculate that gma-miR166u regulates the cold response of nitrogen-fixing nodules by modulating these transcription factors, allowing the signaling pathways to turn on/off. miR2111 has been reported to regulate plant responses to phosphate and nitrogen starvation by targeting Kelch domain-containing F-box proteins in *Arabidopsis* and soybean [62,63,64,65]. Here, we showed that miR2111f was also induced by low temperature treatment in mature nodules of soybean (Figure 2), and two Kelch-related genes (*glyma19g25770* and *glyma16g06160*) were validated as targets of gma-miR2111f in cold-treated nodules (Figure 3 and Figure 4). These Kelch-related proteins share high homology with a Kelch domain-containing F-box protein that functions in the long-distance regulation of the *Lotus*-*Rhizobium* symbiosis (Takahara *et al.*, 2013). It is likely that gma-miR2111 modulates soybean-rhizobia symbioses and stress adaptations. In addition, we found that gma-miR171p may regulate the cold response of nodules through the direct cleavage of a target gene (*glyma11g17490*) encoding a GRAS family transcription factor (Figure 4), although both predicted genes were confirmed to be targets of gma-miR171p (Figure 3). GRAS family transcription factors have been found to be essential for rhizobial Nod factor-induced transcription in rhizobial nodulation and mycorrhizal symbioses [66,67,68]. Moreover, five predicted target genes of miR169c were annotated as HAP2-like transcription factors (Table 1), and one of them (*glyma02g35190*) was validated as a direct target of gma-miR169c in cold-treated nodules (Figure 3 and Figure 4). In many plants, miR169 targets *HAP2*, which encodes a HAP2 transcription factor subunit and a component of the hetero-trimeric CCAAT-box-binding factor complex (CBF/NF-Y/HAP), which binds the CCAAT motif present in many eukaryotic promoters [69]. *MtHAP2-1*, which encodes a transcription factor belonging to the NFY-A family and which is targeted by miR169, has been shown to be required for nodule development [70]. However, the targeting of *HAP2*-like genes by miR169 has not been reported. It is likely that *HAP2*-like genes function as targets of miR169 to regulate cold responses (*i.e*., nitrogenase activity) in nodules.

Nitrogen fixation is a complex trait that is precisely and dynamically controlled by a complicated molecular network. Our results indicate that the transcription of cold-responsive miRNAs and their target genes may be controlled by cold-responsive transcription factors and transcriptional activators/repressors involved in auxin signaling (Figures S2 and S3). Our results provide novel insight into the regulatory networks underlying the nodular response to low temperatures. Functional analyses of miRNAs/targets and their signaling pathways, and of the molecular mechanisms of miRNA regulation in response to low temperatures, will help us to decipher the molecular mechanisms of nitrogen fixation efficiency control under low-temperature conditions. Because the majority of legumes are warm climate plants and nodulation and nitrogen fixation control are highly conserved in legumes, our research has important implications for the elucidation of nitrogen fixation efficiency control at low temperatures.

## 3. Experimental Section

### 3.1. Plant Materials and Low Temperature Treatment

Seeds of soybean (*G. max* cv. Williams 82) were surface-sterilized by treatment with 70% ethanol for 1 min, followed by 10% bleach for 4 min and then rinsed five times with sterile deionized water. The sterilized seeds were planted in a 6-inch pot filled with a 3:1 (*v*/*v*) mixture of sterilized vermiculite and perlite. The pots were pre-wetted with a low nitrogen plant nutrient solution and placed into a controlled growth chamber (24‒26 °C, 200 μmol·m^−6^·s^−6^, 16-h/8-h day/night photoperiod). The seedlings were watered every five days with the same low nitrogen solution. For rhizobium inoculation, *B. japonicum* strain USDA110 was grown in tryptone/yeast extract (TY) medium, centrifuged and resuspended in sterile deionized water to an optical density at 600 nm of 0.08. Five-day-old seedlings were flood-inoculated with the *B. japonicum* suspension culture (30 mL per plant).

For measurement of the Lb content and nitrogen use efficiency in mature nodules, seedlings inoculated with *B. japonicum* 28 days earlier were rinsed briefly in phosphate-buffered saline (PBS, pH 7.5) to remove the vermiculite and perlite particles and transferred to low nitrogen solution. The seedlings were chilled (CH) at 4 °C or left untreated (CK) for 24 h prior to analysis, and then, the nodules were collected. The harvested nodules were immediately frozen in liquid nitrogen for RNA extraction.

### 3.2. Analysis of the Leghemoglobin (Lb) Concentration

A pyridine-hemochrome assay was used to determine the Lb concentration [71]. Fresh nodules were collected and macerated with 2 mL of PBS. Nodular debris was discarded after filtration through two layers of cheesecloth. The filtrate from the last step was centrifuged at 10,000× *g* for 15 min. Next, 2 mL of the extract (suitably diluted) were mixed thoroughly with an equal volume of alkaline pyridine reagent. A few crystals of sodium were added to the greenish-yellow solution to reduce the hemochrome. The resulting solution was stirred without aeration, and the absorbance was read at 556 nm after 2 min against plain reagent as a blank using a NanoDrop 2000c spectrophotometer (Thermo Fisher Scientific, Waltham, MA, USA). The Lb concentration was estimated as described by Appleby and Bergersen [71].

### 3.3. Analysis of the Acetylene Reduction Activity (ARA) Level

After treatment, mature nodules (28 days post-inoculation (dpi)) were collected from the treated plants, and the ARA level was analyzed by gas chromatography, as described previously [36]. The mature nodules were collected in a 25-mL serum bottle sealed with an isobutyl rubber stopper. A syringe was used to replace 10% of the air in the bottle with the equivalent amount of C_2_H_2_, and the bottle was placed in the dark at 28 °C for 2 h. An Agilent gas chromatograph system (Agilent 7890 A, Agilent Technologies, Wilmington, DE, USA) was used to measure the ratio of C_2_H_2_ to ethane in the bottle (the parameters were as follows: KB Alumina capillary column, 30 m; column temperature, 80 °C; detector temperature, 200 °C; carrier gas, N_2_, air and H_2_; and purity of the standard gas (C_2_H_2_), >99.9%). The ARA of nitrogenase was calculated using the following formula [72]:

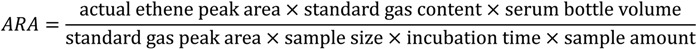
(1)

### 3.4. Small RNA Library Construction, Solexa Sequencing and Data Analysis

For small RNA library construction and high-throughput sequencing, plants with mature nodules at 28 dpi were exposed to 4 °C for 24 h. Small RNAs were isolated from the treated (CH) and untreated nodules (CK) with a mirVana™-miRNA Isolation Kit (Ambion, Austin, TX, USA), followed by sequencing using an Illumina-Solexa 1 Genetic Analyzer at the Beijing Genomics Institute (Beijing, China). The read counts for miRNAs in the two libraries were normalized to one million (normalized expression = actual miRNA count/total count of clean reads × normalized one order of magnitude).

Data analysis was done as described by Dong [47]. Contaminating sequences, including adaptors, insert tags, sequences shorter than 18 nt or longer than 30 nt and those that could not be aligned with soybean genomic sequences were removed from the raw data. A BLAST analysis (E-value ≤ 0.01) was executed to exclude sequences matching exons and introns of mRNAs, rRNAs, tRNAs, scRNAs and snoRNAs in the NCBI GenBank database [73] and Rfam database [74]. The sequences filtered by the procedure described above were used for the following analysis.

### 3.5. Quantitative Reverse Transcription PCR (RT-qPCR)

Small RNAs were isolated from low temperature-treated or untreated soybean nodules using a mirVana™-miRNA Isolation Kit (Ambion) according to the manufacturer’s instructions. First-strand cDNA synthesis was performed using a miRcute miRNA first-strand cDNA synthesis kit with an oligo (dT) adaptor primer (5'-GCGAGCACAGAATTAATACGACTCACTATAGGT (A, G, or C) (A, G, C, or T)-3'). Quantitative real-time PCR was performed using an ABI PRISM 7500 Real-Time PCR System (ABI, Foster City, CA, USA) as follows: the reaction mixture (20 μL) contained 10 μL of SYBR Premix Ex Taq (Takara Bio Inc., Otsu, Japan), 2 μL of first-stand cDNA, 0.2 μM each primer, 0.4 μL of ROX Reference Dye II and 6.8 μL of ddH_2_O. The PCR mixtures were preheated at 95 °C for 30 s, followed by 40 cycles of amplification (95 °C for 5 s and 60 °C for 34 s). The forward primers used for the selected miRNAs and the reverse primer containing the adaptor sequence are included in Table S3. Our RT-qPCR data were analyzed using the software built into the 7500 system (ABI, Los Angeles, CA, USA). Each experiment contained three biological replicates.

For expression validation of the target genes, total RNA samples isolated using an RNase Plant Mini Kit (Tiangen Biotech, Beijing, China) were processed. Reverse transcription reactions were performed at 55 °C with 5 μg of RNA using SuperScript III Reverse Transcriptase (Invitrogen, Carlsbad, CA, USA). Oligo (dT) primers were used as reverse transcription primers according to the manufacturer’s instructions. The PCR primers used are included in Table S4. Real-time PCR was carried out using an ABI PRISM 7500 Real-Time PCR System (ABI), and SYBR Premix Ex-Taq (Takara Bio Inc.) was used to detect the PCR products. The reactions were incubated in a 96-well plate at 95 °C for 5 min, followed by 40 cycles of 95 °C for 5 s and 60 °C for 30 s. All reactions were run in triplicate.

### 3.6. miRNA Target Gene Prediction and Experimental Validation

Candidate miRNA target genes were determined using publicly available prediction algorithms, including psRNAtarget [43] and the target search tool in WMD3 [44]. Known *G. max* open reading frames downloaded from the soybean Genome Annotation Database (Phytozome) [75] were used for miRNA target prediction. To select putative miRNA-target pairs, only four mismatches between the mRNA targets and miRNAs were allowed [22].

For experimental validation of the putative target genes, total RNA was extracted from mature nodules and polyA mRNAs were isolated using a PolyATract^®^ mRNA Isolation System III (Promega Corp., Fitchburg, WI, USA). PolyA mRNAs were then subjected to 5'-RACE assays using a GeneRacer Kit (Invitrogen), omitting the calf intestinal phosphatase and decapping reaction according to the manufacturer’s instructions. For each candidate target, two reverse gene-specific primers were designed. Nested PCR was performed to amplify the cleaved transcripts with reverse primers and GeneRacer adaptor-specific primers. The specific primers used for the putative targets are shown in Table S5. The PCR products were subsequently cloned into pMD19-T (Takara Bio Inc.) for sequencing.

## 4. Conclusions

SNF has been studied for a long time, but little is known about the molecular mechanism of SNF regulation under cold conditions. Sequencing analyses of soybean and other legumes have allowed researchers to investigate this issue. In this study, we showed the genome wide expression profiles of miRNAs in response to chilling in soybean nodules, described several miRNAs involved in the response of mature nodules to cold treatment and validated their target genes. miRNA-mediated gene regulation of the targets was confirmed to be part of this process in this tissue. Therefore, our results shed light on the roles of miRNAs in protection against chilling temperatures in soybean nodules. Functional analysis of miRNAs and their targets in response to low temperatures will help us to understand the molecular mechanisms by which nodules can cope with chilling injury and maintain a high nitrogen fixation rate in soybean under low temperatures.

## Supplementary Files

Supplementary File 1
