# Supplementary material for: Identification of Cold-Responsive miRNAs and Their Target Genes in Nitrogen-Fixing Nodules of Soybean
Source: Int J Mol Sci. 2014 Aug 5;15(8):13596–614. doi: 10.3390/ijms150813596 (PMC4159813; doi:10.3390/ijms150813596)
Supplement: Supplementary File 1 [file ijms-15-13596-s001.pdf]

Supplementary Information

Figure S1. Length distribution and abundance of the sequence. Histogram presentation of sequence-length distribution for clean reads in two libraries (CK: untreated; CH: cold-treated). The *x*-axis indicates sequence sizes from 9 to 31 nt. The y-axis indicates the percent of reads for every given size. The histogram shows there are two peaks in small RNAs profile in soybean.

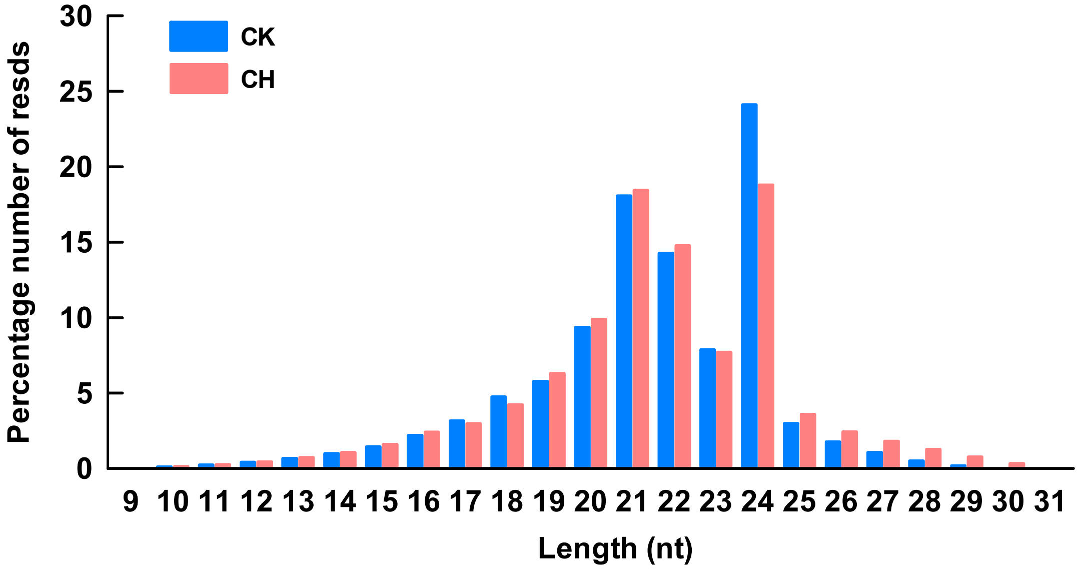

Figure S2. Analysis of miRNAs promoter. The promoter sequences upstream of pre-miRNAs (2000 bp) were chosen as promoter sequences [75] for analyzing cis-elements using software [42].

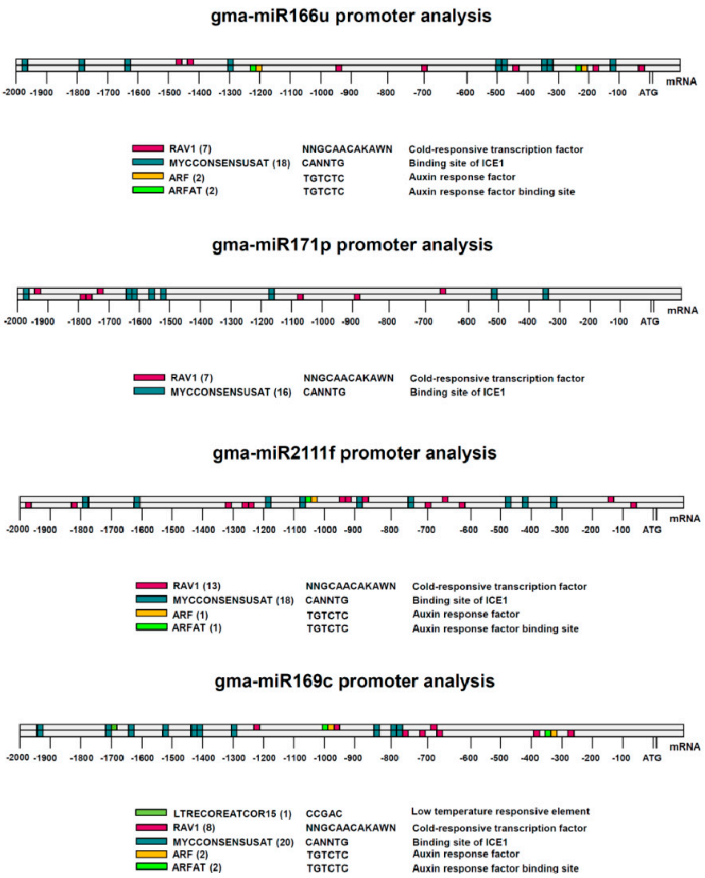

Figure S3. Promoter analysis of target genes. The 2000 bp sequences upstream of start codon "ATG" of the genes were chosen as promoter sequences [75] for analyzing *cis*-elements using software [42].

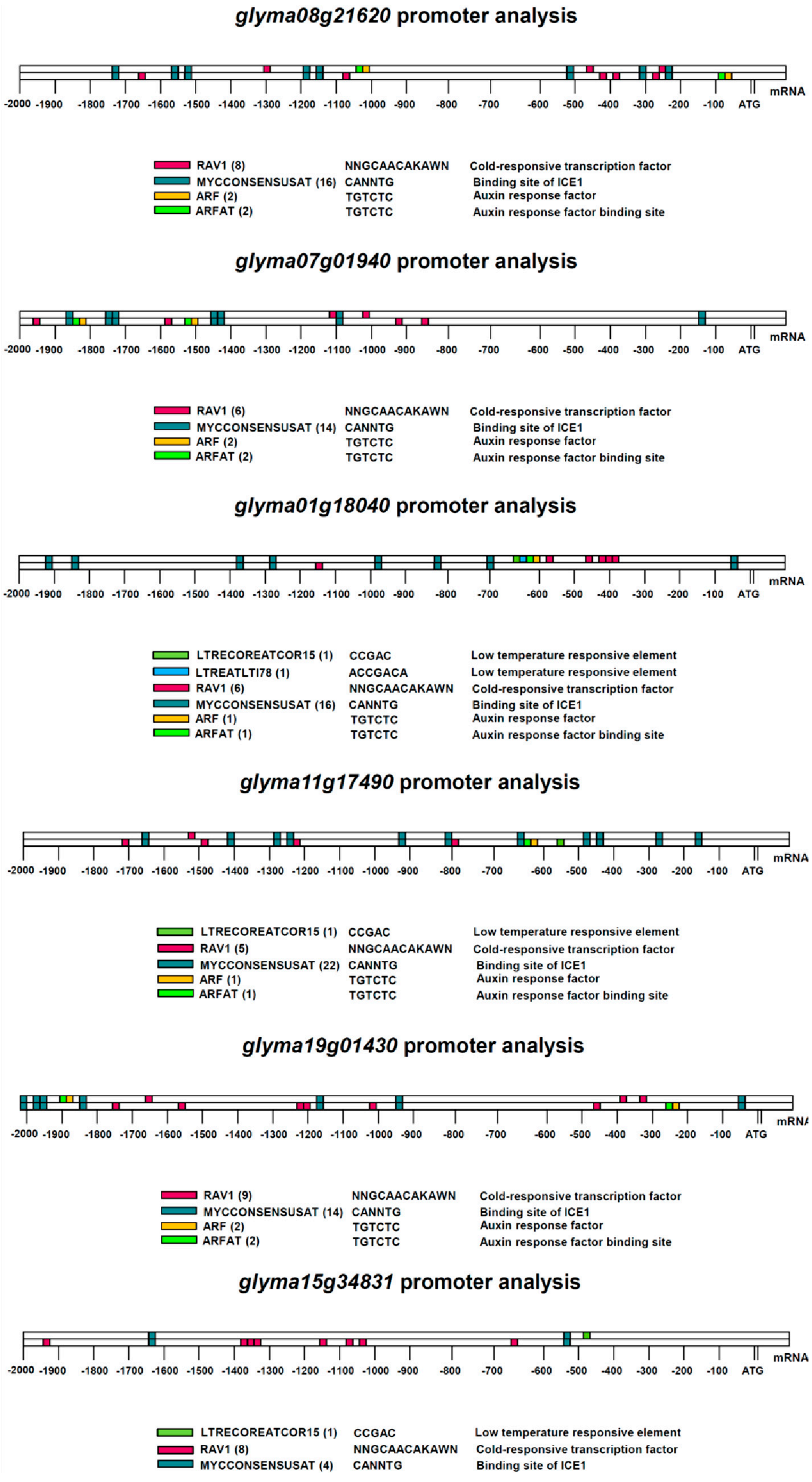

Figure S3. *Cont.*

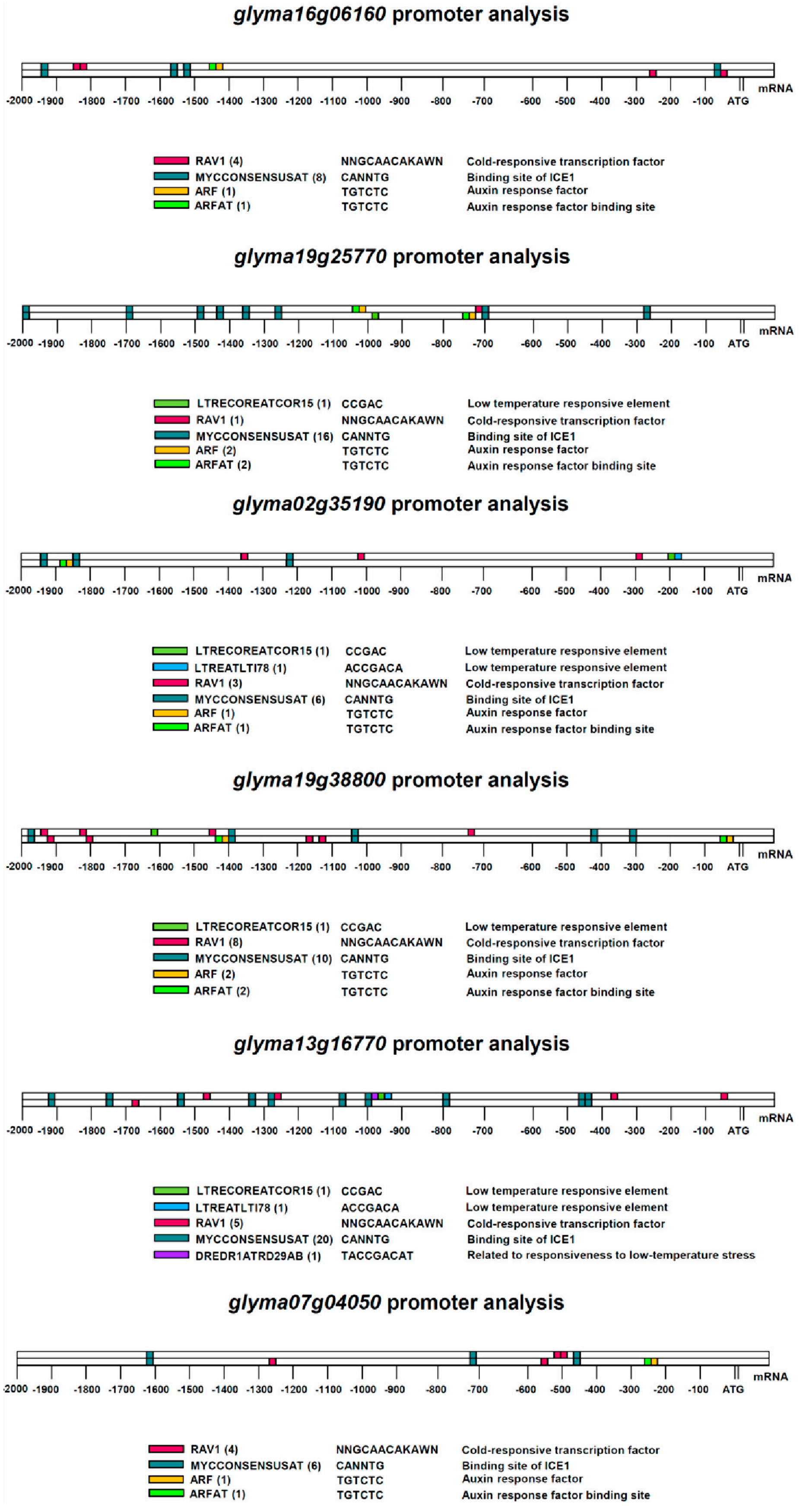

Table S1. Solexa sequencing result of the abundance and different expression of miRNAs in CK (untreated) and CH (cold-treated) libraries.

Expression Pattern	Name	CK	CH	Fold-Change (CH/CK)
Up-Regulated	gma-miR166a	31,122	103,293	3.32
	gma-miR482	303	977	3.22
	gma-miR166b	37,119	114,129	3.07
	gma-miR390a-3p	106	420	3.96
	gma-miR167a	21,623	38,467	1.78
	gma-miR167b	21,628	38,465	1.78
	gma-miR167d	21,628	38,465	1.78
	gma-miR167e	19,557	34,122	1.74
	gma-miR167f	19,557	34,122	1.74
	gma-miR167c	23,629	39,957	1.69
	gma-miR390a-5p	1494	2490	1.67
	gma-miR168	10,116	15,767	1.56
	gma-miR390b	17	27	1.59
	gma-miR1515	184	282	1.53
	gma-miR1510a	3463	5086	1.47
	gma-miR396b	1748	2546	1.46
	gma-miR396c	1748	2546	1.46
	gma-miR396a	3197	4299	1.34
	gma-miR1507a	133,760	179,146	1.34
	gma-miR1507b	133,760	179,146	1.34
	gma-miR164	1112	1472	1.32
	gma-miR172a	7257	9422	1.30
	gma-miR172b	7257	9424	1.30
	gma-miR171b	4824	6010	1.25
	gma-miR1510b	2904	3543	1.22
	gma-miR162	944	1060	1.12
	gma-miR172c	5291	5962	1.13
	gma-miR172d	5291	5962	1.13
	gma-miR172e	5291	5962	1.13
	gma-miR398a	235	249	1.06
	gma-miR398b	235	249	1.06
	gma-miR156b	258	267	1.03
	gma-miR1509b	62,260	63,212	1.02
	gma-miR171p	12	86	7.17
	gma-miR166u	43	190	4.42
	gma-miR399i	376	1594	4.24
	gma-miR2111f	80	151	1.89
	gma-miR397a	862	1373	1.59

Table S1. *Cont.*

	Name	CK	CH	Fold-Change (CH/CK)
**Down-Regulated**	gma-miR159c	335	20	0.06
	gma-miR169c	68	4	0.06
	gma-miR159b	158	16	0.10
	gma-miR319a	20	5	0.25
	gma-miR319b	20	5	0.25
	gma-miR160	459	165	0.36
	gma-miR169b	34	16	0.47
	gma-miR2109	7974	3808	0.48
	gma-miR1523	37	18	0.49
	gma-miR2119	778	456	0.59
	gma-miR156e	391,003	241,747	0.62
	gma-miR156c	393,772	244,826	0.62
	gma-miR156d	395,059	245,386	0.62
	gma-miR1520d	442	312	0.71
	gma-miR1511	28,100	19,928	0.71
	gma-miR156a	204,878	161,777	0.79
	gma-miR159a	10,096	8540	0.85
	gma-miR393	148	127	0.86
	gma-miR1535	315	279	0.89
	gma-miR169a	22	20	0.91
	gma-miR1509a	127,333	127,248	1.00
	gma-miR5037	31	13	0.42
	gma-miR5559	489	74	0.15

Table S2. Statistics of CK and CH sRNA libraties from soybean mature nodule.

Category	Library Name	
	CK	CH
Total reads	16,885,324	17,062,436
High quality reads	13,708,461 (100%)	14,075,568 (100%)
Reads smaller than 18 nt	1,267,915 (9.25%)	1,360,690 (9.67%)
Clean reads	12,303,456 (89.75%)	12,541,791 (89.10%)
Total sRNA reads mapping to genome *	7,607,757/12,303,456 (61.83%)	7,234,067/12,541,791 (57.68%)
Unique sRNA reads mapping to genome *	2,294,582/5,350,950 (42.88%)	1,775,562/5,038,749 (35.24%)

* Total sRNA reads and unique sRNA reads were mapped to the *Glycine max* genome v1.0 sequences available in Phytozome [75].

Table S3. List of primers for qPCR analysis of miRNAs.

Primer Name	Primer Sequence (5'-3')
5.8s rRNA Real-Forward Primer	ACGCCTGCCTGGGTGTCACAC
RT-Reverse Primer	GCGAGCACAGAATTAATACGACT
gma-miR397a Real-Forward Primer	TCATTGAGTGCAGCGTTGATG
gma-miR166u Real-Forward Primer	ACCAGGCTTCATTCCCCA
gma-miR167c Real-Forward Primer	TGAAGCTGCCAGCATGATCTG
gma-miR169c Real-Forward Primer	CCAAGGATGACTTGCCGA
gma-miR319a/b Real-Forward Primer	TTGGACTGAA GGGAGCTCCC
gma-miR5037a Real-Forward Primer	GCCTCAAAGGCTTCCACTACTG
gma-miR1523a Real-Forward Primer	ATGGGATAAATGTGAGCTCAAAA
gma-miR2111f Real-Forward Primer	TAATCTGCATCCTGAGGTTTAAAA
gma-miR171p Real-Forward Primer	TTGAGCCGCGTCAATATCTTA
gma-miR399i Real-Forward Primer	CCAAAGGAGAGCTGCCCTG
gma-miR5559 Real-Forward Primer	TACTTGGTGAATTGTTGGATCAAA

Table S4. List of primers for qPCR analysis of target genes.

Primer Name	Primer Sequence (5'-3')
Glyma01g18100-Real-F	ATGAAGGCCGTGCCCCTA
Glyma01g18100-Real-R	TAACCCTCCTTGAGGTGGGTT
Glyma11g17490-Real-F	CCAGATGTCCTTCAGATGATTGAG
Glyma11g17490-Real-R	AACACTCAGCCTGGGATTCTG
Glyma02g35190-Real-F	GGGTAATAAGTCGTTGTTTGGAGG
Glyma02g35190-Real-R	AGGACTTCATTTGACCACAATACTCC
Glyma07g01940-Real-F	GATTACTACGCAGGCAACCAAAG
Glyma07g01940-Real-R	CAGCAGTCCCAGTAGCCTTAGAA
Glyma06g21620-Real-F	CGGAATGGATAACGGGAAGTAC
Glyma06g21620-Real-R	CTGGAACCAAACCTTGATTTGC
Glyma16g06160-Real-F	CATAGTTCCCAAAAGAAGATGAGAGTTA
Glyma16g06160-Real-R	GCCAAGATTGAAGTCTCTAGCTCATC
Glyma19g25770-Real-F	GCAATGGTTCCTCTACTAATGAAGATG
Glyma19g25770-Real-R	TGAGAGAGCCAAGAACCGCTT

Table S5. Primer sequence for target genes 5'RACE validation.

Primer	Sequence
Glyma08g21620-IR	CAACAACCAGAAGTCTCGAGCG
Glyma08g21620-OR	GATCAACAATGTGGATGATGGAACC
Glyma07g01940-IR	GCTCAATGGTTCCACCATTTGC
Glyma07g01940-OR	AGGTACCCACTAGGCAGCATCTCA
Glyma01g18040-IR	GTGAAATTGGCGAACTGCAGAAC
Glyma01g18040-OR	CCATTCCTGAGTGCTAACTCTTGC
Glyma11g17490-IR	TTAGCGAACTGCAGAACTGGTGAG
Glyma11g17490-OR	CCATTCCTGAGTGCTAACTCTTGC
Glyma16g06160-IR	TCTCCACTTGCCAGCATGAAC
Glyma16g06160-OR	CCATCAATCTCCTTGCCAGAGACT
Glyma19g25770-IR	CACTTACCAGCATGAACACTGAAGGT
Glyma19g25770-OR	CCATCAATCTCCTTGCCTGAGAC
Glyma02g35190-IR	GAAGCCTAAAATGTTAACCACACCC
Glyma02g35190-OR	GTTGTTGTGAGAATGTGATTGCTCTG
